# PTEN Inhibition in Human Disease Therapy

**DOI:** 10.3390/molecules23020285

**Published:** 2018-01-30

**Authors:** Rafael Pulido

**Affiliations:** 1 Biomarkers in Cancer Unit, Biocruces Health Research Institute, 48903 Barakaldo, Spain; rpulidomurillo@gmail.com; 2 IKERBASQUE, Basque Foundation for Science, 48013 Bilbao, Spain

**Keywords:** small molecule PTEN inhibitor, neuroregeneration, tissue injury, wound healing, response to infection, infertility, stem-cells, cancer, diabetes, pain relief

## Abstract

The tumor suppressor PTEN is a major homeostatic regulator, by virtue of its lipid phosphatase activity against phosphatidylinositol 3,4,5-trisphosphate [PI(3,4,5)P3], which downregulates the PI3K/AKT/mTOR prosurvival signaling, as well as by its protein phosphatase activity towards specific protein targets. PTEN catalytic activity is crucial to control cell growth under physiologic and pathologic situations, and it impacts not only in preventing tumor cell survival and proliferation, but also in restraining several cellular regeneration processes, such as those associated with nerve injury recovery, cardiac ischemia, or wound healing. In these conditions, inhibition of PTEN catalysis is being explored as a potentially beneficial therapeutic intervention. Here, an overview of human diseases and conditions in which PTEN inhibition could be beneficial is presented, together with an update on the current status of specific small molecule inhibitors of PTEN enzymatic activity, their use in experimental models, and their limitations as research or therapeutic drugs.

## 1. Introduction

**Figure 1:**
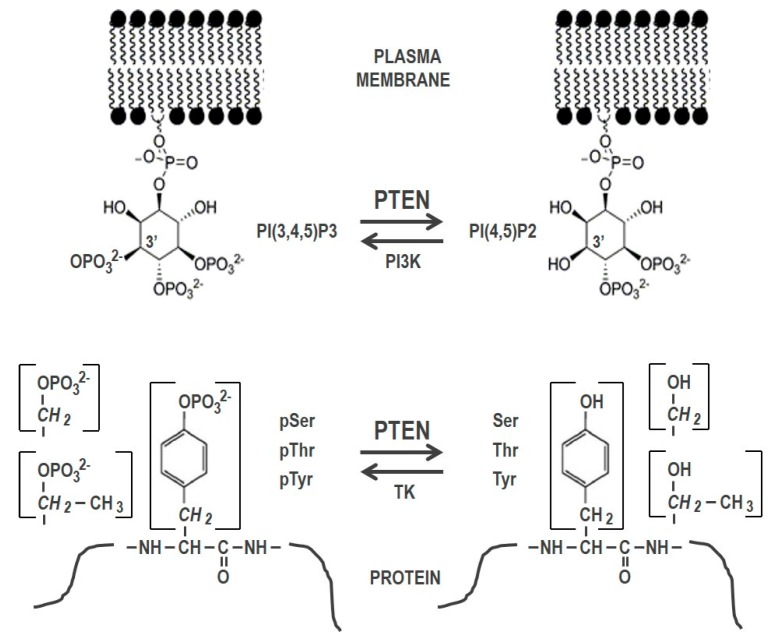
Schematic depiction of PTEN generic substrates. In the upper panel, dephosphorylation of PI(3,4,5)P3 at the plasma membrane is shown. In the bottom panel, protein dephosphorylation is shown, with the side chain of Ser, Thr, or Tyr amino acids in brackets. PI3K, phosphatidylinositol-4,5-bisphosphate 3-kinase; TK, tyrosine kinase.

PTEN is a highly conserved dual-specificity protein tyrosine phosphatase (PTP) of ubiquitous expression, with capacity to dephosphorylate both inositol lipids and proteins (Figure 1). PTEN protein presents an N-terminal PTP catalytic domain followed by a C2-lipid-binding domain, which are essential for the stability and function of the enzyme and for the association of PTEN to membranes. At the C-terminus, PTEN possesses a disordered C-terminal tail with regulatory functions. Several lipid- and protein-binding motifs, distributed at different PTEN regions, facilitate PTEN recruitment to the proximity of substrates at several subcellular compartments [1,2]. Recently, PTEN isoforms with extended N-termini and common PTP and C-terminal domains, which distribute at different intracellular and extracellular locations, have been described, and their physiologic expression and function are under scrutiny [3]. 

A distinctive feature of PTEN, in comparison with other PTPs, is the presence of a wider and negatively charged catalytic pocket with capability to accommodate both phospho-inositol lipids and phospho-amino acids, which explains in part PTEN substrate specificity [4]. PTEN dephosphorylates in vivo the lipid second messengers phosphatidylinositol 3,4,5-trisphosphate [PI(3,4,5)P3] and phosphatidylinositol 3,4-bisphosphate [PI(3,4)P2] to produce phosphatidylinositol 4,5-bisphosphate [PI(4,5)P2] and phosphatidylinositol 4-phosphate [PI(4)P], opposing the action of the class I PI3Ks [5,6,7]. This unique activity makes PTEN a major homeostatic regulator and tumor suppressor protein, whose function is absent or defective in a wide variety of tumors as a result of somatic alterations.

Moreover, *PTEN* gene is mutated with relatively high frequency in the germline of PHTS (PTEN Hamartoma Tumor Syndrome) and Macrocephaly/Autism Syndrome patients [8,9,10]. A role for PTEN as a inositol 1,3,4,5,6-pentakisphosphate [I(1,3,4,5,6)P5] phosphatase has also been proposed, although it is not clear whether this activity is physiologically relevant [11,12,13] (Table 1). PTEN protein phosphatase activity has been reported towards a variety of membrane bound, cytoplasmic, and nuclear protein substrates, although in some cases it is uncertain whether these are *bona fide* PTEN direct substrates (Table 1). It has been proposed that the major physiologic effect of PTEN protein phosphatase activity is its autodephosphorylation at the C-terminal region [14,15]. This would restrain PTEN intramolecular interactions, regulating its subcellular location and modulating positively its lipid phosphatase activity [14,16,17,18]. Defined mutations at the PTEN active site have rendered PTEN variants with specific loss of lipid- or protein-phosphatase activity [19,20,21]. These PTEN variants are currently used as instrumental tools in the laboratory to delineate the catalytic requirements of the diverse PTEN biological activities. However, the differential physiologic regulation of PTEN lipid- and protein-phosphatase activities is unknown, and the identification of inhibitors that only affect one of these activities, but not the other, is not documented.

Regulation of PTEN expression levels, protein conformation, and subcellular localization, is achieved by a variety of genetic, epigenetic, postranscriptional, and postranslational mechanisms affecting PTEN gene, mRNA, and protein [1,2,22]. Pathologic alterations of these mechanisms may cause a decrease in PTEN protein, or PTEN mislocalization, which associate with cancer predisposition [23,24]. In addition, PTEN catalysis is regulated in vivo by intracellular redox, a process which differentially affects to distinct groups of Cys-based PTPs [25,26,27,28]. In particular, the PTEN Cys124 catalytic residue at the active site is highly sensitive to oxidation and forms a disulfide bond with the Cys71 residue in the presence of reactive oxygen species (ROS), which permits reversible inactivation of the enzyme [29]. Several redox-effector proteins, including glutathione [30], peroxiredoxin 1 (Prdx1) [31], thioredoxin 1 (Trx1) [26,32], and glutaredoxin 5 (Grx5) [33] have been proposed to reactivate PTEN catalysis in cells by reducing its oxidized form.

## 2. Small Molecule PTEN Inhibitors

**Figure 2:**
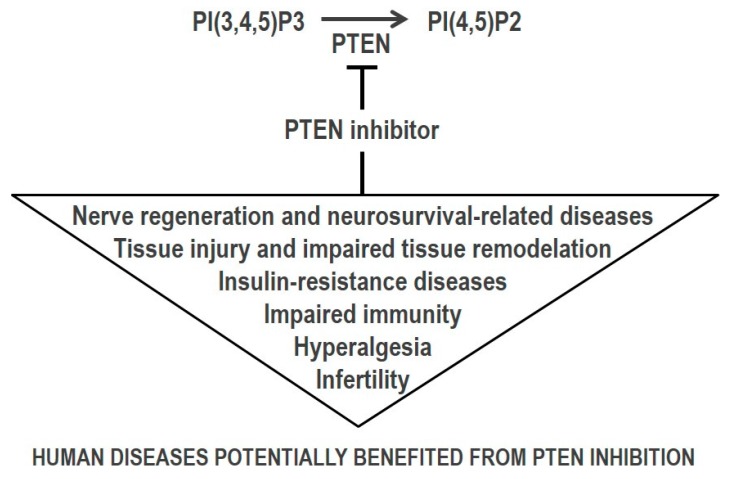
Significance of PTEN inhibition in human disease therapy. Human diseases which could benefit from PTEN pharmacologic inhibition are indicated (see text for a more comprehensive description). Note that the potential beneficial effect of PTEN inhibition has been related with PTEN PI(3,4,5)P3 lipid phosphatase activity. Whether PTEN inhibition may be beneficial in some conditions in relation to PTEN protein phosphatase activity needs to be explored.

The overall positive role of the PI3K/AKT/mTOR signaling pathway in cell growth and survival makes the rational for the therapeutic targeting of PTEN-deficient cancers with PI3K, AKT, or mTOR small molecule inhibitors [56]. Alternatively, reconstitution of PTEN expression or activity, when feasible, could also decrease PI3K-mediated oncogenic signaling, with potential therapeutic benefit in PTEN-deficient cancers [57,58]. On the other hand, in physiologic cell regeneration processes, or in conditions associated to neurodegeneration, tissue injury or ischemia, as well as in insulin resistance metabolic disorders, increased signaling through the PI3K/AKT/mTOR pathway is needed, making inhibition of PTEN catalysis a potential pharmacologic intervention (Figure 2) [59,60,61]. The potential side effects of inhibiting the catalytic activity of PTEN tumor suppressor are manifest. This prompts to methodologies (not addressed in this review) that specifically interfere with the distinct PTEN biological activities, without targeting directly PTEN catalytic active site, as suitable alternatives to PTEN catalysis inhibition.

Vanadium and peroxovanadium compounds are extensively used as general inhibitors of protein tyrosine phosphatases [62,63,64]. More in particular, bisperoxovanadium compounds, including bpV(phen) (bisperoxovanadium 1,10-phenantroline), bpV(pic) (bisperoxovanadium 5-hydroxipyridine), bpV(HOpic) (bisperoxovanadium 5-hydroxipyridine-2-carboxylic acid), bpV(pis) (bisperoxovanadium pyridin-2-squaramide), as well as the related vanadium complex VO-OHpic (hydroxyl(oxo)vanadium 3-hydroxypiridine-2-carboxylic acid) have been proposed as reversible and relatively specific small molecule PTEN inhibitors, with IC50 values in the nM range [65,66,67,68] (Table 2). PTEN inhibition by bpV(phen) is caused by the formation of Cys124-Cys71 oxidative disulfide-bridged PTEN species, which can be reverted by reducing agents, as it happens with the inhibition of PTEN by ROS [26,69]. In addition, a phenanthrenedione-related compound, SF1670 (*N*-(9,10-dioxo-9,10-dihydrophenanthren-2-yl)pivalamide), is also currently being used experimentally at the low μM range as a relatively specific PTEN inhibitor [70,71] (Table 2), although the analysis of SF1670 to target selectively PTEN has not been fully addressed. Therapeutic use of these compounds is not only challenged by their potential negative influence in cancer prevention, but also for their likely additional selectivity towards other PTPs under physiologic conditions [72,73,74,75]. This makes necessary further deep investigation before the current PTEN inhibitors can be safely used in human therapy. Table 3 shows a list of diseases and conditions in which pharmacologic inhibition of PTEN by these small molecule inhibitors has been experimentally explored as a potential therapeutic approach.

## 3. PTEN Inhibition by Small Molecules in Human Disease Therapy

### 3.1. Nerve Regeneration and Neurosurvival-Related Diseases

Downregulation of signaling through the axis PI3K/AKT/GSK-3β/mTOR by neuronal PTEN restrains axon outgrowth and nerve regeneration in peripheral and central nervous systems, both during embryonic development and after neuronal injury or ischemia. In addition, PTEN plays an important role in limiting the synaptic function in the signaling through NMDA and AMPA receptors. This makes inhibition of PTEN a feasible molecular approach to revert neurological damage under pathological circumstances [139,140,141,142,143,144].

The stimulation of nerve growth and regeneration after physical, ischemic, or diabetic damage to brain or peripheral neurons constitute one of the more feasible PTEN inhibition-based therapies. In line with the reports by Park et al. on the promotion of axon regeneration after optic nerve injury in PTEN retinal ganglion cells conditional knockout mice [145], and by Liu et al. on the positive effect on axon regrowth following spinal cord injury in corticospinal neurons PTEN-deleted mice [146], many animal studies support the AKT/GSK-3β/mTOR-dependent negative role of PTEN in nerve growth and regeneration [147,148,149,150,151,152,153,154,155,156]. Accordingly, pre-injury and post-injury treatment with PTEN-inhibiting bpV compounds has shown to be beneficial in several rat models of brain-, spinal cord-, and sensory neurons-traumatic injury (Table 3, and references therein). Combination therapy using bpV(pic) and Schwann cell transplantation further improved motor neuron repair after cervical contusive spinal cord injury in rats [83]. In line with drug-combination treatments, GSK-3β, a downstream effector in PTEN-mediated signaling, is also a pro-apoptotic enzyme that inhibits neuroprotection upon neuronal injury [85,157]. It would be interesting to test the potential additive effect in neuronal survival and regeneration of inhibiting pharmacologically both PTEN and GSK-3β.

PTEN expression and subcellular compartmentation are altered in the brain from Alzheimer’s disease (AD) patients [158,159,160,161], and the involvement of PTEN in regulating tau phosphorylation and amyloidβ (Aβ) peptide-induced neurotoxicity in human cell lines and mouse primary neuron cultures has been documented [93,95,161,162]. In this context, a physical association between tau and PTEN in N1E115 human neuroblastoma cells has been described, which resulted in restraining of PTEN lipid phosphatase activity [163]. bpV(pic) treatment of SH-SY5Y human neuroblastoma cells resulted in inhibition of okadaic acid-induced tau phosphorylation [93], as well as in protection to Aβ (25-35)-induced toxicity [96]. In addition, VO-OH(pic) treatment of APP/Psen transgenic mice, a mouse model of AD, rescued synaptic function and mouse cognitive deficits, in parallel with protecting from Aβ (49)-induced NMDAR-dependent long term depression and synaptic toxicity events, a phenomena which was dependent on PTEN recruitment to synaptic spines through PTEN-PDZ interactions [94,164]. In line with a positive role for PTEN in facilitating AD-related neurodegeneration, APP/Psen transgenic mice were found to display increased PTEN expression in the hippocampus, and treatment with bpV(HOpic) decreased apoptosis of hippocampus neuronal cells [95].

Mice with conditional deletion of *Pten* gene in neurons, oligodendrocytes (OLGs), or glial cells display hypermyelination, which is accompanied, in some models, by progressive myelin sheath abnormalities and white matter degeneration [165,166,167,168]. Furthermore, OLG PTEN-deleted mice challenged with lysolecithin injection into the spinal cord white matter, a model of CNS demyelination, did not show improvement in myelin repair [167]. In contrast, it has been reported that combination of bpV(phen) and insulin-like growth factor-1 (IGF-1) promotes myelination in rat and human OLG progenitors cultures [97], suggesting a potential therapeutic application of bpV(phen) in multiple sclerosis (MS). Whether bpV compounds are effective pro-myelinating agents in in vivo models needs to be addressed. In this regard, cerebellar granule cells (GC) PTEN-deleted mice displayed an expanded population of OLG progenitors, with enhanced OLG differentiation and de novo myelination [169], whereas antigen presenting cells (APCs) PTEN-deleted mice displayed protection to inflammatory demyelinating experimental autoimmune encephalomyelitis (EAE) [170]. Further studies are necessary to delineate the physiologic role of PTEN in the different stages of myelination and the potential benefit of PTEN inhibition in myelination-related disorders therapy.

Long-term cognitive and learning dysfunctions are associated with repeated exposure of infants to anesthesia, in association with neurotoxicity and deficits in neurogenesis and neural precursor cells self-renewal [171]. In a neonatal propofol-exposure mice model, PTEN expression was increased while phospho-AKT decreased in dorsal hippocampus, and administration of bpV(phen) reverted the decrease in hippocampal long-term potentiation and long-term memory [98]. Similarly, bpV(pic) administration in a postnatal isoflurane-exposure rat model resulted in improvement in learning and memory performance, in parallel with the restoration of the PSD-95/NMDAR synaptic function and attenuation of tau phosphorylation [100].

It has been reported the neuroprotective effect of bpV(pic) in a hippocampal-excitotoxic mouse model of acquired temporal lobe epilepsy (TLE) triggered by intraperitoneal injection of kainic acid, in parallel with an increase in phospho-AKT levels. Interestingly, PTEN accumulated in the mitochondria from hippocampal cells following kainic acid treatment of mice, an event that was delayed in mice treated with bpV(pic) [101]. This could suggest a positive role for mitochondrial PTEN in mediating TLE-related neuronal excitoxicity. On the other hand, total or partial loss-of-function mutations at the *PTEN* gene are frequent in the germline of patients with Cowden disease, one of the major manifestations of PHTS, and several cases of patients with Cowden disease associated to epilepsy have been reported [172,173,174,175,176]. This suggests that impaired PTEN function may favor epilepsy episodes, in agreement with the notion of using inhibitors of the mTOR PTEN downstream effector as antiepileptic drugs [177]. Whether PTEN inhibition may be therapeutically beneficial in human epilepsy demands further investigation.

Finally, bpV(pic) also restored phospho-AKT levels and attenuated apoptosis in hippocampal developing neurons in an infant rat model of pneumococcal meningitis [102].

### 3.2. Ischemia/Reperfusion Tissue Injury

Ischemia/reperfusion (I/R)-associated diseases constitute one of the most frequent causes of death in humans, mainly due to the instrumental role of I/R on myocardial infarct and stroke. Tissue damage is elicited by the lack of oxygen and nutrients supply during the ischemic period and it is exacerbated after tissue reoxygenation, which triggers a ROS-mediated damaging and proinflammatory response [178]. Since signaling through the PI3K/AKT/mTOR pathway is an important protection mechanism against I/R injury, especially in the case of ischemic conditioned cardiac tissue, PTEN inhibition has been proposed as a suitable therapeutic intervention in I/R cardiac damage [179]. In line with this, PTEN protein degradation and oxidative-inactivation were increased in perfused rat hearts during ischemia preconditioning, in parallel with augmented phospho-AKT levels and cardioprotection [180]. In addition, PTEN +/− mice display improved protection induced by ischemic preconditioining and attenuated inflammation and myocardial remodeling after myocardial infarction [181,182], whereas deletion of *Pten* gene in cardiomyocytes results in cardiac hypertrophy and cardiac contractility dysfunction, as well as in protection to pathological hypertrophy in response to pressure overload [183,184,185]. Thus, full inhibition of PTEN in muscle cardiac cells may be causative of pathology, although could be beneficial after acute episodes of cardiac damage. Interestingly, PTEN deletion in myeloid cells was protective for liver I/R injury by increasing the non-inflammatory M2 macrophage population [186].

The work of Keyes et al. testing the effect of bpV(HOpic) in rat cardiomyocytes subjected to I/R [103] was followed by additional studies using different PTEN inhibitors and I/R mechanical and chemical rat and mouse models (Table 3, and references therein). Overall, treatment with bisperoxovanadium and vanadium derivatives resulted in protection to I/R cardiac injury and improvement of cardiac functions. Importantly, I/R hepatic injury was also attenuated by bpV(HOpic) administration in rat hepatocytes I/R models [110], whereas PTEN inhibition by bpV(HOpic) or bpV(pic) aggravated renal dysfunction and triggered tubular damage in acute I/R and cisplatin/induced kidney injury mouse models [187,188]. This illustrates how tissue-specificity may be relevant in the potential implementation of PTEN inhibitors in human disease therapy.

### 3.3. Wound Repair and Tissue Maintenance

Wound healing responses include polarized endothelial cell migration accompanied by cell survival and proliferation, which are positively regulated by the PI3K/AKT/mTOR pathway. Accordingly, PTEN activity has been associated with delayed wound healing in several human cell types and rodent models, including lung epithelial injury-, muscle regeneration-, skin healing-, or gastric mucosa integrity-models, among others [189,190,191,192,193]. Interestingly, opposite sensitivity responses to acute lung injury have been found in lung epithelial PTEN-deleted mice depending on the lung region affected [191,194]. This should be taken into consideration for the application of lung injury PTEN inhibition-based therapeutic interventions.

The beneficial effects of different bpV compounds on lung, vascular and corneal epithelium injury-, skin renewal-, muscle regeneration-, and retinal detachment-models have been reported (Table 3, and references therein). Remarkably, in vitro studies with epithelial human cells have revealed that enhanced cell migration accounts for the majority of wound repair upon PTEN inhibition with bpV(phen) [112]. Since PTEN protein phosphatase activity plays a major role in the regulation of cell migration [195], an attractive approach for wound healing and tissue repair therapies could be the use of protein phosphatase activity-specific PTEN inhibitors.

### 3.4. Response to Infection

A negative role for PTEN in host defense phagocytic functions, including decreasing chemotaxis, recruitment to inflamed sites, and Fcγ-R-mediated responses, has been evidenced using ex vivo macrophage cell models and myeloid cells PTEN-knockout mice [119,196,197,198]. This sustains the potential use of PTEN inhibitors to improve or restore the phagocytic immune response after bacterial infection, especially in cases of impaired innate immune response or immune system-depletion therapies. In this context, bpV(pic) treatment of rat alveolar macrophages (AM) was beneficial to restore the phagocytic response to sheep red blood cell challenging under conditions of prostaglandin E2-mediated Fcγ-R inhibition [119]. Similarly, AM from bone marrow transplanted mice with impaired lung host defense against Pseudomonas aeruginosa showed restored phagocytosis upon bpV(pic) treatment [120]. Finally, Li et al. has reported the favorable use of SF1670 compound to enhance the efficacy of granulocyte transfusion upon neutropenia-related infections, both in terms of granulocyte recruitment to inflamed sites and phagocytic functions [71].

### 3.5. Infertility

The work with oocyte PTEN-deleted mice indicates that PTEN is essential to limit the activation of primordial ovarian follicles, but not of growing developed follicles, and that lack of PTEN function may be causative of premature ovarian failure (POF) [199,200]. Also, ovarian granulosa cells PTEN-deleted mice displayed enhanced ovulation and fertility [201]. This suggests potential benefits for PTEN inhibition in in vitro fertility interventions. In fact, transient treatment of neonatal mouse ovarian follicles with bpV(pic) or bpV(HOpic) resulted in improved maturation of oocytes, which generated healthy and fertile progeny after in vitro fertilization and embryo transfer [121,122]. Short-term treatment with bpV(pic) of fresh or cryopreserved human ovarian tissue was also beneficial to enhance the in vitro activation of primordial follicles and the efficacy of fertility preservation [127]. Furthermore, treatment of human ovarian follicles from POF patients with a combination of bpV(HOpic) and 740 Y-P PI3K activator, followed by autotransplantation, in vitro fertilization, and embryo transfer, was positive for successful reproduction in some patients [124,125]. However, it has also been reported that human ovarian follicles in culture treated with bpV(pic) or bpV(HOpic) displayed limited growth and reduced survival [126,202]. In addition, deletion of PTEN in ovarian theca cells resulted in aberrant androgenesis and early fertility loss, reminiscent of human polycystic ovary syndrome [203]. Thus, transient low-dose inhibition of PTEN during in vitro manipulation of ovarian cells arises as a suitable approach to improve in vitro fertilization techniques.

### 3.6. Stem Cells-Based Therapy

PTEN plays a crucial and tissue-specific role in stem cell maintenance and self-renewal, with important manifestations in development and tumorigenesis [204,205]. PTEN-knockdown human embryonic stem cells (hESCs) display increased self-renewal, survival, and proliferation, as well as aberrant differentiation potential manifested as a bias toward neuroectoderm differentiation [206]. In mammalian spermatogonial stem cells, PTEN knockdown resulted in higher Nanog expression [207], and a role for PTEN in cell reprogramming of human and mouse fibroblasts has also been proposed based on the enhancement of generation of induced pluripotent stem cells (iPSCs) upon knockout or down-regulation of PTEN expression [128,208]. Furthermore, transient inhibition of PTEN by bpV(HOpic) in mouse embryonic fibroblasts (MEFs) enhanced the production of three-layer germline-competent iPSCs upon ectopic expression of OKSM or OKS factors, in association with accelerated cell proliferation during the early phase of reprogramming [128]. It would be necessary to test whether these observations can be extended to human fibroblasts. Finally, treatment of human bone marrow-derived stromal cells (hBMSC) with SF1670 increased osteogenic differentiation in vitro, in agreement with the findings that osteoblast PTEN-knockout mice displayed increased bone mineralization and improved healing of bone fracture [209,210]. This suggests a potential benefit for PTEN inhibition in conditions where bone mineralization and regeneration needs to be enhanced.

### 3.7. Cancer-Related Diseases

In spite of its role as a major tumor suppressor in many types of cancer, some studies have highlighted the possibility that PTEN pharmacologic inhibition may be an anticancer therapeutic approach under certain circumstances. It has been shown that in prostate PTEN-deleted mice, as well as in PTEN-deleted MEFs or PTEN-deficient human glioblastoma cells, a p53-dependent senescence program is triggered (PTEN-loss-induced cellular senescence, PICS) that restricts tumorigenic processes [130,211,212]. It has also been reported that, upon VO-OHpic treatment, cell senescence is induced and xenograft tumorigenic growth is decreased in *Pten* +/− MEFs or in human prostate or hepatocarcinoma cancer cells expressing reduced levels of PTEN [130,131]. Of relevance, the cell viability inhibitory effect of VO-OHpic in Hep3b hepatocarcinoma cells in vitro was synergistic with the inhibitory effect of drugs targeting VEGFR, MEK, or PI3K/mTOR oncogenic kinases [131]. This opens the possibility that the combined treatment of PTEN inhibitors with currently used targeted therapies could be beneficial in PICS-competent cancers.

A role for PTEN in the suppression of the anticancer immune response has been proposed based on the findings that mice with PTEN deleted in regulatory T-cells (Tregs) are more prone to tumorigenesis and inflammation due to a deficient immunosuppressive T-cell activity in the tumor microenvironment. Furthermore, VO-OHpic treatment of mice with established melanoma or lymphoma tumors resulted in the induction of an inflammatory antitumor response [132], suggesting that pharmacological inhibitory targeting of PTEN could add benefits to anticancer immunotherapies. However, it has also been reported that PTEN deficiency in Tregs causes an autoimmune-lymphoproliferative and proinflammatory disease, as a result of the expansion of T helper-cell populations [213,214]. In addition, PTEN deletion in tumor melanoma cells inhibited T-cell mediated anticancer responses, in line with the notion that PTEN expression in melanoma tumors correlates with higher immune surveillance responses [215]. Together, these observations indicate that a transient and cell-specific PTEN inhibition would be necessary to achieve benefits in the context of anticancer immunotherapies.

Finally, treatment of the BON human carcinoid cell line with VO-OHpic reduced serotonin secretion, which is symptomatic of carcinoid syndrome that occurs secondary to a fraction of carcinoid tumors [133]. Since reduced expression of PTEN in BON cells after stable transfection of PTEN-shRNA resulted in higher metastatic potential in nude mice [216], the potential therapeutic use of PTEN inhibitors to ameliorate carcinoid syndrome has important limitations. It should be tested whether acute but transient pharmacologic PTEN inhibition reduces morbidity associated to carcinoid syndrome without exacerbating carcinoid tumors.

### 3.8. Insulin-Resistance Metabolic Diseases

PTEN PIP3-phosphatase activity counteracts insulin signaling, making PTEN inhibition a potential therapeutic approach for type 2 diabetes [61,217,218]. Although systemic long-term PTEN pharmacological inhibition is likely to be harmful, it is possible that a tissue-specific intervention or a partial inhibition of PTEN might be beneficial in type 2 diabetes treatment. For instance, skeletal muscle, pancreatic β-cells-, or adipose tissue-PTEN-knockout mice displayed protection to insulin resistance and type 2 diabetes, without malignant cell growth or cancer manifestations [219,220,221,222]. Likewise, diminishing systemically PTEN levels with antisense oligonucleotides reverted insulin resistance and hyperglycemia in diabetic mice [223]. PTEN-antisense treatment of C2C12 mouse myoblasts abolished TNFα-induced insulin resistance, which was associated with increased PTEN mRNA and protein levels [224]. Furthermore, PTEN increased expression in TNFα-treated H-411E rat liver cells was reverted by treatment with VO-OHpic [134]. Importantly, VO-OHpic treatment of mouse mesenteric arteries challenged with TNFα, or treatment of arteries from mice fed with high fat diet, prevented the induction of vascular insulin resistance [136]. These findings suggest that PTEN inhibitors could prevent insulin resistance upon pathological circumstances. Further mechanistic studies are required to understand the regulation of PTEN expression in insulin-responsive tissues by TNFα and upon PTEN catalysis inhibition.

### 3.9. Pain Relief/Antinociception

A role for PTEN in regulating hyperalgesia and nociception has been proposed based on the observations that animals from rat and mice models of migraine displayed improved mechanical thresholds and less spinal trigeminal neuron activation upon PTEN knockdown, in association with diminished tyrosine phosphorylation of the of the NMDA receptor NR2B subunit [225,226]. Accordingly, the increased mechanical allodynia shown by rats injected with NMDA was attenuated upon co-injection with bpV(phen), with concomitant decrease in Src-mediated NR2B phosphorylation [137]. In addition, PTEN has been involved in the negative regulation in sensory neurons of surface expression of δ opioid analgesic receptors (δR). Treatment of PC12 rat pheochromocytoma cells or mouse trigeminal neurons with bpV(phen), bpV(HOpic), or SF1670 promoted the surface delivery of newly synthesized δR, which resulted in increased antihyperalgesic efficacy of δR upon agonist stimulation [138]. These findings suggest that pharmacological PTEN inhibition could be beneficial in attenuation of chronic pain processes or in analgesic interventions using δR agonists. However, in a rat neuropathic model of chronic constriction injury, downregulation of spinal cord PTEN was associated with nociception [227], suggesting that PTEN activity could also support antinociceptive functions. Further work is required to ascertain the role of PTEN in pain modulation under physiological and pathological conditions.

## 4. Concluding Remarks

Important efforts have been made to develop specific and biologically active small molecule PTEN inhibitors, and to validate them as suitable experimental and clinical tools. This has provided compounds targeting PTEN that are widely used in research, as well as the proof-of-concept that PTEN pharmacologic inhibition could be beneficial for human welfare in specific diseases or conditions. Although currently available PTEN inhibitors are commonly used in many studies as specific and potent PTEN inhibitors, evidence exists that most of these compounds target, at least, several PTPs, and that their potency is dependent on the redox experimental conditions [75]. This makes convenient the refinement in the obtaining and characterization of PTEN inhibitors, both in terms of potency and target selectivity. Examples exist that some bpV compounds frequently used as PTEN inhibitors may cause beneficial pathology-related effects with independence of PTEN inhibition, but likely dependent on inhibition of another PTP. For instance, injection of bpV(phen) in a murine model of allergic asthma resulted in decreased allergic and lung inflammatory responses [228], although a plethora of in vitro and in vivo studies support the notion that PTEN activity mitigates asthmatic features [229,230,231,232,233]. Promiscuous specificity does not necessarily accompany bad therapeutic outcomes for a given inhibitory compound, as illustrated with the current therapeutic use in clinical oncology of small molecules inhibiting several tyrosine kinase targets. As far as toxicity and side effects are limited, it is possible that multiple targeting helps the favorable therapeutic outcome of some current PTEN inhibitors. Thus, it has been proposed the added potential benefit to confer neuroprotection of the PTEN-partially selective bpV(pis) inhibitor by targeting both PTEN and the ERK1/2 MAPK pathway [68].

Regarding the use of current PTEN inhibitors in research, some aspects should be taken into consideration to endorse the assumption that the observed experimental effects of the compounds are, in fact, due to PTEN inhibition. These include, among others, dose-response analysis using more than one inhibitory compound; the analysis of pAKT content as an indirect readout of PTEN inhibition; the comparative analysis of both PTEN-positive and PTEN-negative cells; and the comparative analysis of the compound with PTEN-knockdown or PTEN dominant-negative strategies, if feasible.

Most of the pathologic conditions in which PTEN inhibition is proposed as a potential therapeutic approach rely on the direct negative control by PTEN PIP3-phosphatase activity on the signaling through the PI3K/AKT/mTOR pathway (Figure 2). However, evidence exists on the importance of PTEN protein-phosphatase activity, as well as non-catalytic PTEN activities, on physiologic and pathologic processes [195,234]. Whether selective inhibition by small molecules of PTEN lipid- or protein-phosphatase activity is achievable needs to be explored. In addition, since PTEN catalytic activity is involved in feedback loops that regulate PTEN expression, it would be interesting to test the effects of current PTEN inhibitors in non-catalytic PTEN functions, such as those exerted in the cell nucleus.

The current knowledge of the outcomes of PTEN pharmacologic inhibition discloses a wide scenario of possibilities for therapeutic intervention. In summary, acute local treatment with suboptimal doses of PTEN inhibitors, rendering tissue- or organ-specific tuned PTEN inactivation at well-defined frame times, could circumvent undesired side effects of chronic or total PTEN inactivation, and set the conditions for the beneficial use of PTEN inhibition in human disease therapy.

## Figures and Tables

**Table 1:** Physiologic/potential PTEN substrates ^1^.

Substrate	Functional Outcome of Dephosphorylation	References
*Phosphatydilinositol/Inositol substrates (3′ specificity)*		
PI(3,4,5)P3, PI(3,4)P2 ^2^	Inhibition of PI3K/AKT/mTOR pathway	[6,7,34]
I(1,3,4,5,6)P5 ^3^	Inhibition of cell proliferation	[11,12,13]
*Protein substrate* ^4^		
AKT (Thr)	AKT inactivation	[35]
b-catenin (Tyr)	Maintenance of cell-cell adhesion	[36]
Cofilin-1 (Ser)	Activation of actin depolymerization	[37]
CREB (Ser)	CREB inactivation	[38]
Drebrin (Ser)	Changes in neuronal actin dynamics	[39]
EphR (Tyr) (*C. elegans*)	EphR inactivation	[40]
FAK (Tyr)	Inhibition of directional cell migration	[41]
FYN (Tyr)	FYN inactivation	[42]
IR (Tyr)	IR inactivation	[43]
IRF3 (Ser)	Inhibition of IRF3 nuclear import	[44]
IRS1 (Tyr)	Inhibition of insulin and IGF signaling	[45]
MCM2 (Ser)	Inhibition of replication fork progression	[46]
PDGFR (Tyr)	PDGFR inactivation	[47]
PTEN (Ser, Thr)	PTEN conformational opening and activation	[14,15,48]
PTK6 (Tyr)	PTK6 inactivation	[49]
p85b (Tyr, Thr)	AKT inactivation	[50]
Plk1 (Thr)	Plk1 inactivation	[51]
Rab7 (Tyr, Ser)	Promotion of late endosomal maturation	[52]
SHC (Tyr)	Inhibition of random cell migration	[53]
SRC (Tyr)	SRC inactivation	[54]
5-HT2cR	5-HT2cR inactivation	[55]

^1^ Updated from reference [1]; ^2^ PI(3,4,5)P3 is the more physiologically relevant PTEN phosphatydilinositol substrate; ^3^ Physiological dephosphorylation of I(1,3,4,5,6)P5 by PTEN is under debate; ^4^ Potential protein substrates (not validated as PTEN direct substrates in all cases). Dephosphorylation of Ser, Thr, or Tyr, is indicated in brackets. AKT, AKT/PKB kinase; CREB, cAMP response element-binding protein; EphR, Eph receptor; FAK, focal adhesion kinase; FYN, FYN kinase; IR, insulin receptor; IRF3, interferon-regulatory factor 3; IRS1, insulin receptor substrate-1; MCM2, maintenance complex component 2; PDGFR, platelet-derived growth factor receptor; Plk1, polo-like kinase 1; PTK6, protein tyrosine kinase 6; Rab7, Rab GTPase 7; SHC, Src homology 2 domain containing-adaptor protein; SRC, SRC kinase; 5-HT2cR, serotonin 5-HT2c receptor.

**Table 2:** PTEN inhibitors ^1^.

Inhibitor	Chemical Name	IC50	Chemical Structure	Reference
bpV(phen)	bisperoxovanadium 1,10-phenantroline	38 nM	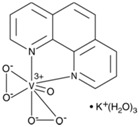	[67]
bpV(pic)	bisperoxovanadium 5-hydroxipyridine	31 nM	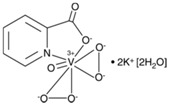	[67]
bpV(HOpic)	bisperoxovanadium 5-hydroxipyridine-2-carboxylic acid	14 nM	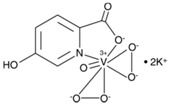	[67]
bpV(pis)	bisperoxovanadium pyridin-2-squaramide	39 nM	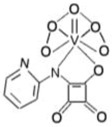	[68]
VO-OHpic	hydroxyl(oxo)vanadium 3-hydroxypiridine-2-carboxylic acid	35 nM	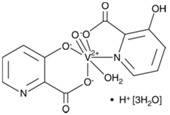	[66]
SF1670	*N*-(9,10-dioxo-9,10-dihydrophenanthren-2-yl) pivalamide	2 μM	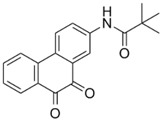	[70]

^1^ Small molecule compounds currently used as direct inhibitors of PTEN catalysis are indicated.

**Table 3:** Selected studies using small molecule PTEN inhibitors with potential therapeutic application.

Disease/Condition	Experimental Model	Inhibitor	Reference
*Nerve regeneration and neurosurvival-related diseases*			
Central nervous system (CNS) and peripheral nervous system (PNS) injury	- Vertebral arteries occlusion ischemia rat model	- bpV(pic)	- [76]
	- 6-hydroxydopamine midbrain injection rat model of dopaminergic neurons degeneration	- bpV(phen)	- [77]
	- Thoracic spinal cord contusion injury rat model	- bpV(phen)	- [78]
	- Carotid artery ligation ischemia postnatal rat model	- bpV(pic)	- [79]
	- Rat sensory neurons; sciatic nerve cut rat model	- bpV(pic)	- [80]
	- Middle cerebral artery occlusion (MCAO) ischemia rat model	- bpV(pic)	- [81]
	- Hemicontusive spinal cord injury (SCI) rat model	- bpV(pic)	- [82,83,84]
	- Cortical neuron oxygen/glucose deprivation (OGD)-rat model cultures	- bpV(pic)	- [85]
	- Fluid percussion-traumatic brain injury rat model	- bpV(pic)	- [86]
	- MCAO ischemia rat model	- bpV(HOpic)	- [87]
	- Hippocampal neuron culture stretch injury rat model	- bpV(HOpic)	- [88]
	- Human embrionic stem cells (hESC)-derived neuronal progenitors	- bpV(pic)	- [89]
	- Subarachnoid hemorrhage (SAH)-early brain injury rat model	- bpV(pic)	- [90]
	- MCAO ischemia rat model	- bpV(phen)	- [91]
	- SH-SY5Y human neuroblastoma cells; cortical neuron OGD rat cultures; MCAO ischemia rat model	- bpV(pis)	- [68]
	- Sciatic nerve transection-regeneration type 2 diabetic mouse model	- SF1670	- [92]
Alzheimer’s disease (AD)	- SH-SY5Y human neuroblastoma cells	- bpV(pic)	- [93]
	- App/Psen1 Tg mice model of AD	- bpV(HOpic), VO-OHPic	- [94]
	- App/Psen1 Tg mice model of AD	- bpV(HOpic)	- [95]
	- SH-SY5Y human neuroblastoma cells	- bpV(pic)	- [96]
Multiple sclerosis (MS)	- Rat newborn and human fetal olygodendrocyte (OLG) progenitors; rat dorsal root ganglion neuron cultures	- bpV(phen)	- [97]
Cognitive dysfunction associated to postnatal anesthesia exposure	- Neonatal propofol-exposure mice model	- bpV(phen)	- [98]
	- Rat hippocampal neural precursor cells	- bpV(phen)	- [99]
	- Postnatal isoflurane-exposure rat model	- bpV(pic)	- [100]
Epilepsy	- Temporal lobe epilepsy mouse model	- bpV(pic)	- [101]
Pneumococcal meningitis (PM)-induced neuronal death	- Infant rat model of PM	- bpV(pic)	- [102]
*Ischemia/reperfusion (I/R) tissue injury*			
Ischemia/reperfusion (I/R) cardiac injury; myocardial infarct	- Rat cardiomyocytes; carotid artery occlusion I/R rat model	- bpV(HOpic)	- [103]
	- Isolated mouse hearts; carotid artery occlusion I/R mouse model	- VO-OHpic	- [104]
	- MCAO ischemia mouse model	- bpV(phen)	- [105]
	- Mouse cardiomyocytes	- VO-OHpic	- [106]
	- KCl-sudden cardiac arrest-resuscitation mouse model	- VO-OHpic	- [107]
	- Streptozotocin-induced type 1 diabetic rats subjected to myocardial I/R	- bpV(HOpic)	- [108]
	- I/R and H_2_O_2_-induced injury models in H9c2 rat cardiomyoblasts	- bpV(phen), bpV(pic)	- [109]
I/R hepatic injury	- Rat hepatocytes, and liver warm I/R rat models.	- bpV(HOpic)	- [110]
*Wound repair and tissue maintenance*			
Lung epithelium injury; acute lung injury	- Primary human upper airway epithelia; BEAS-2B human bronchial epithelium cells	- bpV(phen), bpV(pic)	- [111,112]
	- Oleic acid-induced acute lung injury mice model	- bpV(phen)	- [113]
Skin renewal	- Human foreskin-derived precursors	- bpV(pic)	- [114]
Corneal wound healing	- Human corneal epithelial (HCE) cells; cornea scratch wound rat model	- bpV(pic)	- [115]
Muscle regeneration	- C2C12 mice myoblasts	- bpV(HOpic)	- [116]
Retinal detachment (RD)	- Retinotomy-RD rat model	- bpV(pic)	- [117]
Vascular endotelial injury associated with hyperglicemia	- Human umbilical vein endothelial cells (HUVECs)	- bpV(phen)	- [118]
*Response to infection*			
Pneumonia/deficit in phagocytic response	- Rat alveolar macrophages (AM)	- bpV(pic)	- [119]
	- AM from syngeneic bone marrow (BM) transplantation mouse model	- bpV(pic)	- [120]
Neutropenia-related infections	- Peritonitis and neutropenia mouse models	- SF1670	- [71]
*Infertility*			
In vitro fertilization	- Mouse neonatal ovarian follicles	- bpV(pic)	- [121]
	- Mouse neonatal ovarian follicles	- bpV(HOpic)	- [122]
	- Mouse non-growing oocytes	- bpV(HOpic)	- [123]
	- Human ovarian follicles	- bpV(HOpic)	- [124,125]
	- Human ovarian follicles	- bpV(HOpic)	- [126]
	- Human ovarian cortex	- bpV(pic)	- [127]
*Stem cells-based therapy*			
Generation of induced pluripotent stem cells (iPSCs)	- Mouse embryonic fibroblasts (MEFs)	- bpV(HOpic)	- [128]
Bone regeneration	- Human BM stem cells	- SF1670	- [129]
*Cancer-related diseases*			
Cancers with PTEN low expression	- MDA PCa-2b human prostate cancer cells	- VO-OHpic	- [130]
	- Hep3b human hepatocarcinoma cells	- VO-OHpic	- [131]
Cancers under immunotherapy	- Mice with established melanoma and lymphoma tumors	- VO-OHpic	- [132]
Carcinoid syndrome	- BON human carcinoid cells; carcinoid syndrome mouse model	- bpV(HOpic)	- [133]
*Insulin-resistance metabolic diseases*			
Type 2 Diabetes	- H-411E rat liver cells TNFα-induced insulin resistance model	- VO-OHpic	- [134]
	- Glucosamine-induced insulin-resistant rat skeletal muscle cells	- bpV(HOpic)	- [135]
	- Mouse mesenteric arteries	- VO-OHpic	- [136]
*Pain relief/Antinociception*			
Inflammatory pain; chronic migraine	- NMDA-triggered nociceptive rat model	- bpV(phen)	- [137]
Antinociception mediated by δ opioid receptors	- PC12 rat pheochromocytoma cells, mouse trigeminal ganglia (TG) neurons	- bpV(phen), bpV(HOpic), SF1670	- [138]
